# Supplementary material for: A Comprehensive Assessment of Ultraviolet-Radiation-Induced Mutations in Flammulina filiformis Using Whole-Genome Resequencing
Source: J Fungi (Basel). 2024 Mar 20;10(3):228. doi: 10.3390/jof10030228 (PMC10971301; doi:10.3390/jof10030228)
Supplement: Supplementary file 1 [file jof-10-00228-s001.zip › Supplementary Material S8/KEGG annotation/out/64381550635650.os/KO/out_map/map04110.html]

KEGG PATHWAY: Cell cycle - Reference pathway


|  |  |
| --- | --- |
| **Cell cycle - Reference pathway** |  |

[
Pathway menu
| Organism menu
| Pathway entry
| Show description
| User data mapping
]

Mitotic cell cycle progression is accomplished through a reproducible sequence of events, DNA replication (S phase) and mitosis (M phase) separated temporally by gaps known as G1 and G2 phases. Cyclin-dependent kinases (CDKs) are key regulatory enzymes, each consisting of a catalytic CDK subunit and an activating cyclin subunit. CDKs regulate the cell's progression through the phases of the cell cycle by modulating the activity of key substrates. Downstream targets of CDKs include transcription factor E2F and its regulator Rb. Precise activation and inactivation of CDKs at specific points in the cell cycle are required for orderly cell division. Cyclin-CDK inhibitors (CKIs), such as p16Ink4a, p15Ink4b, p27Kip1, and p21Cip1, are involved in the negative regulation of CDK activities, thus providing a pathway through which the cell cycle is negatively regulated.
Eukaryotic cells respond to DNA damage by activating signaling pathways that promote cell cycle arrest and DNA repair. In response to DNA damage, the checkpoint kinase ATM phosphorylates and activates Chk2, which in turn directly phosphorylates and activates p53 tumor suppressor protein. p53 and its transcriptional targets play an important role in both G1 and G2 checkpoints. ATR-Chk1-mediated protein degradation of Cdc25A protein phosphatase is also a mechanism conferring intra-S-phase checkpoint activation.

|  |  |
| --- | --- |
| Reference pathway | 100% |
